# In Vivo and In Vitro Studies Assessing the Antiviral Efficacy of Double Combinations Against Coxsackievirus B Infection

**DOI:** 10.3390/microorganisms13010199

**Published:** 2025-01-17

**Authors:** Adelina Stoyanova, Simeon Galabov, Vadim Makarov, Angel S. Galabov

**Affiliations:** 1 The Stephan Angeloff Institute of Microbiology, Bulgarian Academy of Sciences, 1113 Sofia, Bulgaria; 2 Research Center of Biotechnology, Russian Academy of Sciences, 119071 Moscow, Russia

**Keywords:** coxsackievirus, antivirals, double combinations, pleconaril, MDL-860

## Abstract

Coxsackievirus B (CVB) infections, ranging from mild to severe diseases, lack specific antiviral treatments, underscoring the need for novel therapeutic strategies. Drug therapy is an important tool for controlling enterovirus infections, but clinically effective drugs do not currently exist, mainly due to the development of drug resistance. Combination therapy with two or more drugs has the potential to successfully inhibit viral infection more effectively than either drug alone as well as delay the development of resistance. This study explores the consecutive alternating administration (CAA) scheme in mice with CVB1 infection, utilizing double antiviral combinations consisting of pleconaril and MDL-860, with guanidine hydrochloride and oxoglaucine. The CAA combinations of pleconaril achieved a survival rate, in infected mice, of up to 59%, while the combinations of MDL-860 showed no significant effects. CAA reduced mortality, prolonged mean survival time (up to 5 days), and mitigated drug resistance compared to monotherapy or simultaneous administration. Monotherapeutic courses and daily administration of double combinations had no effect. Phenotypic characterization using the IC_50_ marker of virus isolates from brain tissue of infected and treated mice was of particular importance for the evaluation of the CAA treatment scheme. The results show increased susceptibility of the virus isolates to the partner compounds in double CAA combinations. In contrast, virus isolates from the monotherapeutic groups manifested a diminished susceptibility to their respective compound, which signals the development of drug resistance. All data obtained prove the potential of the CAA scheme for the development of effective chemotherapy of enterovirus infections.

## 1. Introduction

Coxsackievirus B1 (CVB1) is a significant human pathogen and a member of the *Enterovirus* genus within the *Picornaviridae* family, known for its wide range of clinical manifestations. CVB1 primarily causes mild, self-limiting illnesses such as febrile episodes, hand-foot-and-mouth disease, or respiratory infections. However, in rare cases, it can lead to severe conditions like myocarditis, aseptic meningitis, or neonatal systemic infections, particularly in newborns and children. Epidemiological studies of patients with nervous system disorders demonstrate the presence of infectious virus, its components, or anti-CVB antibodies. Additionally, CVBs have been implicated in the development of type 1 diabetes mellitus (T1DM), asthma, and allergies, further underscoring their clinical significance. Some experimental studies conducted in vitro and in vivo support the potential association between CVBs and idiopathic neurodegenerative diseases such as amyotrophic lateral sclerosis and psychiatric illness. Despite its clinical importance, traditional antiviral therapies for enteroviruses, including CVB1, are limited to supportive care, and no direct antiviral drugs have been approved for clinical use [1,2].

A large number of compounds are effective in vitro [3,4], but in clinical trials the most active antivirals demonstrate modest effects. The fast development of drug resistance is the main problem in the chemotherapy of enteroviral infections, based until now on monotherapy courses [5]. One way to fight resistance is by applying drug combination therapy of new or already existing substances with different mechanisms of action. Combination therapy with two or more drugs has the potential to successfully inhibit viral infection more effectively than either drug alone. It could achieve greater effects and avoid side effects using lower drug concentrations, and thus, they could prevent the rapid development of drug resistance.

To address the challenges associated with treating CVB infections, our team has investigated the efficacy of consecutive alternating application (CAA) of antiviral agents. This experimental strategy aims to disrupt the viral replication cycle by employing antiviral substances in succession. Specifically, we studied the combined effects of selective inhibitors of enterovirus replication using consecutive, alternating (rather than simultaneous) administration of agents within the combination treatment regimen, referred to as the CAA treatment course. Using this approach, double, triple, and quadruple combinations of some of the most extensively studied anti-enterovirus inhibitors have been tested in vivo [6,7,8,9,10,11,12]. The arrangement of compounds within the combination plays a critical role in determining its effectiveness.

The principle underlying the CAA approach is rooted in the dynamic and adaptive nature of viral replication. CVBs, like many RNA viruses, exhibit a high mutation rate, which contributes to their ability to evade immune responses and develop resistance to antiviral treatments. While a single antiviral agent may inhibit a specific stage of the viral life cycle, CVBs can adapt over time, potentially rendering the treatment less effective or entirely ineffective.

The CAA treatment course seeks to mitigate this limitation by employing two or more antiviral agents in a timed, sequential manner. Alternating antiviral agents allows for the simultaneous targeting of different stages of the viral replication cycle, including viral entry, RNA replication, protein synthesis, and viral assembly. This multipronged approach significantly reduces the likelihood of resistance development. By switching agents at regular intervals, the virus is continuously subjected to different inhibitory pressures, limiting the time available for it to adapt and develop resistance. Furthermore, the use of different antiviral substances can stimulate various arms of the immune system, enhancing the body’s natural defenses in combating the infection.

In this study, we present the results of our studies on the effect of CAA against CVB1 in vivo, applying double antiviral combinations consisting of pleconaril and MDL-860, with guanidine hydrochloride and oxoglaucine. The results show that CAA reduced mortality, prolonged mean survival time, and mitigated drug resistance.

## 2. Materials and Methods

### 2.1. Cell Cultures, Virus, and Compounds

Human epithelial type 2 (HEp-2; National Bank for Industrial Microorganisms and Cell Cultures, Sofia, Bulgaria) cells were maintained in DMEM supplemented with 10% fetal bovine serum (FBS; Gibco BRL, Paisley, Scotland, UK) and 100 U/mL penicillin-streptomycin (Pen-Strep; Lonza, Basel, Switzerland). Cells were cultured routinely at 37 °C in a humidified incubator with 5% CO_2_.

Coxsackievirus B1 (Connecticut 5 strain) used for in vivo experiments was propagated through intracerebral passages (0.02 mL/mouse) in newborn albino mice (ICR line). The virus was prepared as a 10% (*w*/*v*) brain suspension in phosphate-buffered saline (PBS) and stored at −20 °C.

Pleconaril was obtained by Dr. Vadim Makarov (Research Center of Biotechnology, Russian Academy of Sciences, Moscow, Russia). The compound 2-(3,4-dichlorophenoxy)-5-nitrobenzonitrile (MDL-860) was acquired from Prof. Gerhard Pürstinger (Institute of Pharmacy, University of Innsbruck, Innsbruck, Austria); both compounds were dissolved in polyethylene glycol 400 (PEG400). Oxoglaucine (1,2,9,10-tetramethoxy-7H-dibenzo[de,g] quinolin-7-one), an aporphinoid alkaloid isolated from *Glaucinum flavum* Cranz (yellow horn poppy), was obtained from Prof. Stefan Philipov (Institute of Organic Chemistry with Centre of Phytochemistry, Bulgarian Academy of Sciences, Sofia, Bulgaria). It was dissolved in a 1:9 (*v*/*v*) solution of dimethyl sulfoxide (DMSO) and saline. Guanidine hydrochloride, provided by Eastman Organic Chemicals (New York, NY, USA), was dissolved in saline solution.

### 2.2. Animal Experiments

ICR random-bred newborn albino mice were obtained from the Experimental and Breeding Base for Laboratory Animals (BAS, Slivnitza, Bulgaria). Each dam was housed in a well-ventilated acrylic cage with free access to water and food. All animal breeding and experimental procedures were performed in compliance with the guidelines of Bulgaria’s Directorate of Health Prevention and Humane Behavior toward Animals.

Newborn mice (approximately 20–30 animals per group) were inoculated subcutaneously (s.c.) with CVB1 at a dose of 20 MLD_50_ (mouse lethal dose 50%). Animals were subjected to monotherapy or a combined treatment course administered consecutively, starting 1 h post-inoculation (day 1) and continuing through day 12 post-inoculation (dpi). The tested groups included monotherapy courses with each compound and the following double combinations: pleconaril and MDL-860 (PM), pleconaril and oxoglaucine (PO), pleconaril and guanidine hydrochloride (PG), MDL-860 and oxoglaucine (MO), and MDL-860 and guanidine hydrochloride (MG).

The arrangement and dosing schedule of the treatment courses are summarized in Table 1. Daily doses of the compounds were selected as optimal, based on published literature and prior experiments conducted in our laboratory [9,10,13]. The in vivo criteria used to evaluate antiviral efficacy included (i) cumulative lethality, (ii) mean survival time (MST), and (iii) body weight of the treated animals.

### 2.3. Virus Susceptibility to Tested Compounds

Brain tissue was collected daily, starting from 4 dpi and continuing to 12 dpi. Viral samples were subsequently isolated.

The viral content in brain isolates was determined using the cytopathic effect (CPE) inhibition test and assessed by the end-point dilution method, as described by Reed and Muench [14].

To determine the susceptibility of virus isolates to each compound in the tested double combinations, the CPE inhibition test was performed. The monolayer of HEp-2 cells seeded in 96-well plates was infected with 100 TCID_50_ of the relevant virus isolate for 1 h at 37 °C. Following the removal of the virus-containing medium, cells were treated with different concentrations of the tested compounds. The cells were incubated for 48 h at 37 °C, and cell viability was assessed using the neutral red uptake assay based on the initial protocol described by Borenfreund and Puerner [15]. The IC_50_ value, defined as the concentration of the compound required to reduce viral yields by 50%, was calculated as described in previous studies [16,17].

### 2.4. Statistical Analysis

Mortality was monitored until 12 dpi. The survival time was defined as the duration from 1 dpi until the day before death. The protection index (PI) was calculated using the equation: PI = [(PC − 1)/PC] × 100, where PC represents the protection coefficient, defined as the percentage of mortality in the placebo group divided by the percentage of mortality in the drug-treated group.

Statistical significance for mean survival time (MST) and IC50 values was assessed using a one-way ANOVA followed by Bonferroni’s post-test. A *p*-value of less than 0.05 was considered statistically significant, as indicated in the figure legends. Additionally, a two-tailed unpaired *t*-test was conducted alongside the one-way ANOVA to highlight differences in susceptibility among virus isolates in the treated groups.

Mouse survival rates between experimental groups were compared using Fisher’s exact test. The differences in mean survival time (MST) and IC50 values for pleconaril, the drug combinations, and the placebo groups were analyzed using one-way ANOVA with Bonferroni’s correction for multiple comparisons.

## 3. Results

### 3.1. Test of Dose Toxicity in Infected and Non-Virus-Infected Newborn Mice Subjected to Therapy

**Figure 1:**
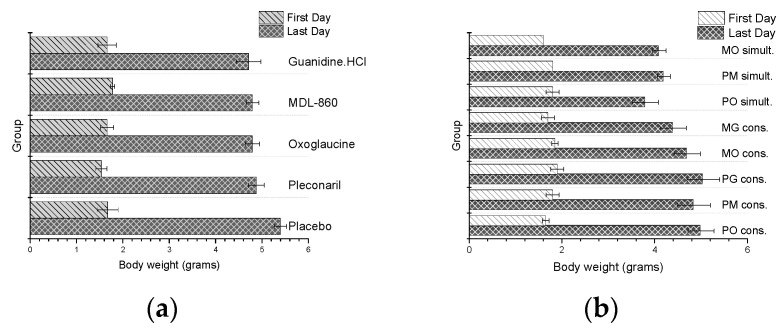
Daily weight of healthy, non-virus-infected animal: (**a**) monotherapy; (**b**) double combinations applied via CAA courses.

To evaluate the toxicity of the double combinations administered during the CAA treatment course, the body weight of the experimental animals was monitored daily. Weights of virus-naive animals treated with dual consecutive or simultaneous combinations, as well as monotherapy courses with the partner compounds, were also reported. The results are presented in Figure 1.

In general, no toxic effects were observed in any of the groups, as evidenced by the absence of early mortality or severe growth retardation in the experimental animals. However, animals treated with co-administered dual combinations exhibited slightly lower body weight compared to other groups.

**Figure 2:**
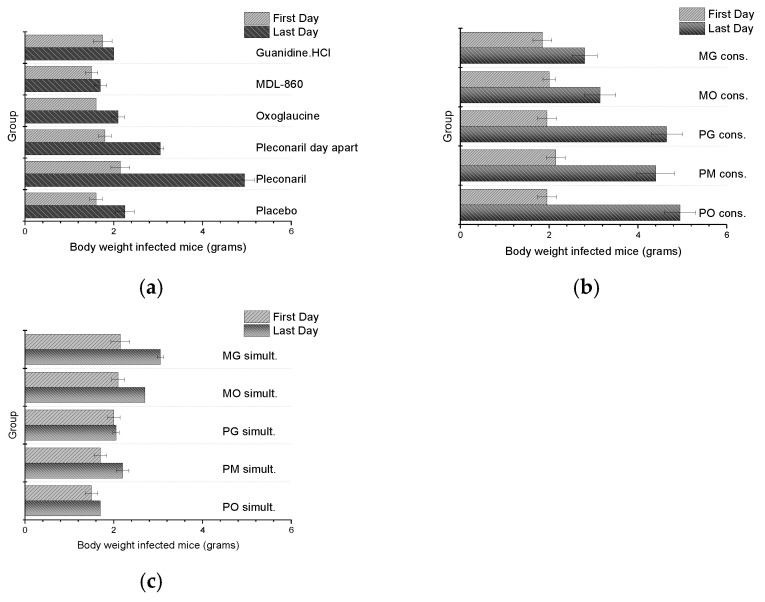
Daily weight of CVB1 Conn-5 infected animals: (**a**) monotherapy; (**b**) double combinations applied via CAA course; (**c**) double combinations applied simultaneously.

The body weights of animals infected with CVB1 Conn-5 (Figure 2) and subjected to dual combination or monotherapy treatments were monitored daily. Despite the potential for the viral infection to influence weight, no significant increase in toxicity was observed in these groups. Early mortality was noted in some groups due to disease progression and lack of therapeutic efficacy.

### 3.2. Antiviral Effects of Combination Therapies Against CVB1 Infection in Newborn Mice

**Figure 3:**
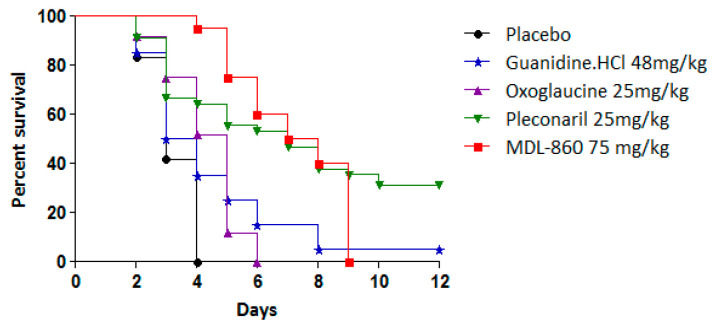
Individual effects of tested compounds against experimental infection with CVB1 in newborn mice.

The antiviral effects of the tested compounds as monotherapeutic agents in an experimental CVB1 infection model are summarized in Table 2 and Figure 3.

Pleconaril exhibited antiviral activity with a protective index (PI) of 26.1%, extending the mean survival time (MST) by 3 days compared to the placebo group. However, when pleconaril was administered every other day, its efficacy was limited, resulting in an MST extension of only 1.5 days. In contrast, MDL-860 demonstrated no protective effects in vivo. Neither oxoglaucine nor guanidine hydrochloride exhibited antiviral activity or extended survival. These findings establish pleconaril as the sole compound with efficacy in this experimental model, although its effects were moderate.

Next, we examined the antiviral effects of the following double combinations applied via the CAA treatment scheme: pleconaril and MDL-860 (PM), pleconaril and oxoglaucine (PO), pleconaril and guanidine hydrochloride (PG), MDL-860 and oxoglaucine (MO), and MDL-860 and guanidine hydrochloride (MG).

The experiments were conducted in newborn mice infected subcutaneously with 20 MLD50 of a neurotropic CVB1 strain within 24 h of birth. The therapeutic course began on the day of infection, one hour post-inoculation, and continued for 12 days post-infection (dpi) or until the animals’ death. Compounds were administered daily in a volume of 0.05 mL per mouse.

**Figure 4:**
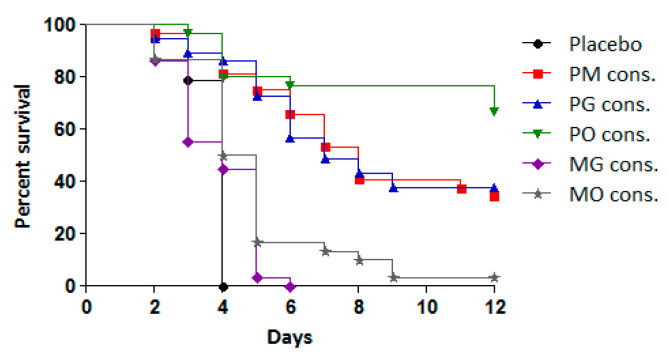
Effect of tested double combinations applied consecutively against experimental infection with CVB1 in newborn mice.

To assess the efficacy of the treatments, the antiviral effects of consecutive administration of double combinations (PM, PO, PG, MO, and MG) were compared to their effects when administered simultaneously at the same time each day. The results are presented in Table 2 and Figure 4.

The combinations PM, PO, and PG administered in the CAA course exhibited significant protective effects compared to the placebo group and the simultaneous administration of the same combinations. This protective effect was reflected in reduced cumulative mortality (PI between 34.4% and 58.8%) and an MST extension of 4–5 days. Among the combinations, PO demonstrated the highest protective effect, with 59% of the infected animals surviving, followed by PG (44%) and PM (34%). All three combinations resulted in a higher PI and MST extension compared to pleconaril monotherapy.

In contrast, the combinations of MDL-860 with either oxoglaucine (MO) or guanidine hydrochloride (MG) showed no significant activity. However, when the MO combination was applied via the CAA scheme, a slight increase in MST was observed compared to the placebo group.

Simultaneous daily administration of all five dual combinations demonstrated no protective effect, with treated animals succumbing to infection within 5–6 days.

### 3.3. Sensitivity to Tested Compounds of Virus Brain Isolates from Mice Subjected to the CAA Treatment Course

The virus progeny in brain samples from mice infected with CVB1 and treated with consecutively applied double combinations or monotherapies were tested for sensitivity to the compounds using the multicycle cytopathic effect (CPE) inhibition assay. A neutral red (NR) uptake method was employed as a colorimetric approach to stain viable cells. Percent protection, defined as the proportion of inhibitor-protected, virus-infected cells retaining viability relative to uninfected cell controls, was calculated using Zhang’s formula [17]. Graphically, the concentration-percentage protection relationship was plotted to determine the inhibitory concentration 50 (IC_50_), representing the compound concentration required to inhibit the virus-induced CPE by 50% [16]. The results are presented in Table 3.

Virus isolates from mice treated with pleconaril alone exhibited a reduced sensitivity to the compound early in the study period, particularly when compared to the placebo group. Over the course of the monotherapy, resistance to pleconaril increased steadily, becoming pronounced toward the end of the treatment. Administering pleconaril every other day also led to a decrease in viral sensitivity, although the differences in sensitivity between the first and last days of the study period were not statistically significant.

In contrast, virus isolates from mice treated with double combinations under the CAA regimen showed a progressive increase in sensitivity to pleconaril. By the final days of the study, sensitivity to pleconaril exceeded that observed in both the placebo group and the monotherapy group. Among the tested combinations, PG demonstrated the highest sensitivity to pleconaril, followed by PO and PM. However, simultaneous administration of the double combinations led to a decrease in pleconaril sensitivity and early death of treated animals, undermining the therapeutic effect observed with the CAA regimen.

The virus isolates from the placebo group at 4 dpi were approximately three times more susceptible to MDL-860 than those from the MDL-860 monotherapy group. In the monotherapy group, resistance to MDL-860 developed rapidly, resulting in highly resistant viral progeny and eventual mortality of the treated animals. Virus isolates from the PM combination under the CAA regimen initially showed low sensitivity to MDL-860, but sensitivity slightly increased by the end of the study period, reaching levels similar to those observed in the placebo group at 4 dpi. In the case of MO and MG combinations administered via the CAA regimen, viral sensitivity to MDL-860 gradually declined over time. Simultaneous administration of these combinations produced rapid resistance development, mirroring the outcomes seen with MDL-860 monotherapy.

The isolates from the placebo group (4 dpi) were about 3-fold more susceptible to MDL-860 than those from the group treated with MDL-860 alone. In the monotherapy group, there was a decrease in susceptibility to the compound and a rapid development of resistant viral progeny accompanied by the death of the treated animals. The virus isolated from the animals treated with PM according to the CAA regimen was less sensitive to the compound in the first days, and the sensitivity increased slightly, and at the end of the study period, it was similar to that observed in the placebo group on 4 dpi of the infection. In the case of the virus isolated from the animals treated with the double combinations of MO and MG according to the CAA scheme, a gradual decrease in the sensitivity of the virus to the compound was observed. The simultaneous administration showed a rapid development of resistance of the viral progeny, comparable to that observed in the monotherapeutic course with MDL-860.

The presence of guanidine hydrochloride in the treatment did not result in significant changes in viral sensitivity during the early days of the study. IC_50_ values remained consistent across most virus isolates tested. By the end of the study, however, the PG combination under the CAA regimen demonstrated a slight increase in viral sensitivity to guanidine hydrochloride compared to the placebo group, while the MG combination had no discernible impact on sensitivity. When administered simultaneously, combinations containing guanidine hydrochloride led to nearly a two-fold reduction in viral sensitivity to the compound, which has limited antiviral efficacy on its own.

Virus isolates from mice treated with oxoglaucine alone displayed lower susceptibility to the compound, with IC_50_ values approximately three times higher than those observed in the placebo group. During the first two days of the study period, viruses isolated from animals treated with double combinations via the CAA regimen showed sensitivity levels similar to those of the placebo group. However, in animals treated with the PO combination via the CAA regimen, viral sensitivity to oxoglaucine increased progressively, achieving IC_50_ values nearly two times lower than those observed in the placebo group on 6 dpi. By contrast, simultaneous administration of oxoglaucine-containing combinations resulted in reduced sensitivity and resistance development in the viral progeny, undermining the therapeutic potential of the compound under these conditions.

## 4. Discussion

The rapid development of drug resistance remains a major challenge in the chemotherapy of enteroviral infections, which until now has relied predominantly on monotherapy courses [5]. Several capsid-binding agents (e.g., pleconaril, disoxaril, pirodavir, vapendavir), 3C protease inhibitors (e.g., rupintrivir), and 3A protein inhibitors (e.g., enviroxime) have failed in clinical trials for human enterovirus infections due to limited efficacy or side effects [18]. Consequently, there is a critical need for more potent therapies and treatments capable of reducing the frequency of drug-resistant virus emergence. One promising strategy is drug combination therapy, which pairs new or existing agents with different mechanisms of action.

The main goals of drug combination therapy are to achieve synergistic therapeutic effects, reduce drug dosages and associated toxicity, and minimize or delay the onset of drug resistance [19]. Experimental approaches using synergistic dual antiviral combinations to combat enterovirus replication have been explored in prior studies [20,21,22,23,24,25,26,27,28,29,30].

This study undertakes a large-scale evaluation of therapeutic courses following the consecutive alternating administration (CAA) scheme, which involves the sequential, non-simultaneous administration of compounds. Five dual combinations of anti-enteroviral agents—pleconaril, MDL-860, guanidine hydrochloride, and oxoglaucine—were tested: PG, PO, PM, MG, and MO. Experimental infection with a neurotropic strain of CVB1 in newborn mice served as the model. Additionally, individual effects and simultaneous daily combinations of the compounds were assessed in vivo.

The results demonstrate that the double combinations of PO, PG, and PM, administered according to the CAA scheme, significantly reduced mortality and prolonged the mean survival time (MST) of treated animals during CVB1 infection. However, the combinations of MG and MO did not exhibit antiviral effects when administered via the CAA scheme.

An important advantage of the CAA approach is its ability to mitigate the potential toxic effects associated with the simultaneous administration of partner compounds. By administering each compound on alternate days, the risk of cumulative toxicity from either individual agents or their combination is minimized. This represents a significant advantage in clinical contexts, particularly for vulnerable patient populations.

No antiviral activity was observed with the simultaneous daily administration of PG, PO, PM, MG, or MO, nor in monotherapy courses with oxoglaucine, MDL-860, or guanidine hydrochloride. In contrast, triple combinations applied via the CAA scheme in our prior studies revealed a high protection index (PI), such as 49% for PMO and 31% for PGO [9,10]. Among the double combinations studied, PO yielded the highest protective effect with 59% survival, followed by PG (44%) and PM (34%). Conversely, combinations starting with MDL-860 lacked significant antiviral activity, highlighting the importance of the order in which agents are administered. Initiating treatment with inhibitors targeting virus entry and uncoating proved most effective.

Both double and triple CAA combinations demonstrated significant MST prolongation compared to placebo groups. For instance, MST increased by nearly 6 days with PMO and by 5 days with PGO. Among double combinations, MST was prolonged by 5 days with PO and approximately 4 days with PM and PG. However, MG and MO combinations failed to significantly extend MST. Phenotypic marker analysis of IC50 values from viral progeny isolated from treated animals revealed a tendency toward reduced viral sensitivity in monotherapy groups, suggesting the development of resistance. In contrast, viral isolates from CAA-treated groups exhibited increased sensitivity to partner compounds, underscoring the scheme’s potential to reduce the risk of resistance development.

Chronic or severe CVB1 infection can require long-term treatment, and prolonged use of a single antiviral can lead to decreased efficacy. Monotherapy often imposes selective pressure that facilitates viral evolution and resistance. In contrast, the CAA approach creates a dynamic therapeutic environment, challenging the virus to adapt to multiple mechanisms of action. This complexity reduces the likelihood of resistance emergence. Furthermore, CAA minimizes cumulative toxicity associated with long-term use of a single drug by alternating agents, which is especially beneficial for chronic or severe infections, such as myocarditis or neonatal CVB infections.

Not all antiviral substances work synergistically, and some combinations may result in adverse interactions. Ongoing research is needed to identify combinations that deliver optimal therapeutic outcomes while ensuring safety. Determining the ideal cycle length for each agent also requires extensive pharmacokinetic and pharmacodynamic studies. Although CAA reduces toxicity associated with prolonged use of a single agent, the long-term safety of alternating multiple drugs must be carefully evaluated. Potential risks, such as cumulative toxicity or adverse interactions, remain areas of concern.

## 5. Limitations

Experiments with mice face several limitations and challenges. Newborn mice are in a stage of rapid growth and development, with immature immune, nervous, and organ systems. They are highly vulnerable, and experimental stress can result in their mortality rate. This makes it difficult to accurately apply the findings to older animals or humans.

## 6. Conclusions

In conclusion, the data confirm the efficacy of the CAA treatment approach in experimental CVB1 infections. The results indicate that consecutive administration of compounds increases viral sensitivity and prevents the emergence of resistant progeny. These findings highlight the potential for developing additional combination therapies for rapidly mutating RNA viruses. By leveraging CAA, new antiviral strategies may emerge, offering more effective treatment options for enteroviral infections and beyond.

## Figures and Tables

**Table 1:** Daily administration of compounds.

**Compounds Used in Combination per Day**												**Groups**	
**12**	**11**	**10**	**9**	**8**	**7**	**6**	**5**	**4**	**3**	**2**	**1**		
M G O G O	P P P M M	M G O G O	P P P M M	M G O G O	P P P M M	M G O G O	P P P M M	M G O G O	P P P M M	M G O G O	P P P M M	PM PG PO MG MO	Consecutive combination (CAA)
Combination of PM, PG, PO, MG or MO is simultaneously applied every day													Simultaneous combination
Each drug partner is applied every day for 12 days													Monotherapy
Saline solution every day													Placebo

Compounds were applied once per day beginning 1 h post-infection (Day 1). P: pleconaril—25 mg/kg per os; M: MDL-860—75 mg/kg sc; G: guanidine-HCl—48 mg/kg sc; O: oxoglaucine—25 mg/kg sc.

**Table 2:** Effects of monotherapy courses and double combinations against CVB1 infection in newborn mice.

**Group**	**Survival/Total ^a^**	**MST ± SD ^b^**	**Mortality %**	**PI %**
Placebo	0/46	4.3 ± 0.7	100.0	0.0
Рleconaril	6/23 *	7.2 ± 0.9 ^^^	73.9	26.1
Рleconaril_day apart_	1/11	5.9	91.7	8.3
MDL-860	0/27	4.7 ± 0.8	100.0	0.0
Oxoglaucine	0/19	4.2 ± 0.8	100.0	0.0
Guanidine.HCl	0/20	4.5 ± 1.1	100.0	0.0
РО consecutively	20/34 ***^, #^	9.7 ± 1.3 ^^^^	41.2	58.8
PG consecutively	14/37 **^, ns^	8.2 ± 1.1 ^^^	56.3	43.8
PM consecutively	11/32 **^, ns^	8.4 ± 1.9 ^^^	65.6	34.4
MO consecutively	1/29	5.5 ± 2.1	96.6	3.4
MG consecutively	0/31	4.6 ± 1.8	100.0	0.0
РО simultaneously	0/10	5.3	100.0	0.0
PG simultaneously	0/11	6.1	100.0	0.0
PM simultaneously	0/10	5.2	100.0	0.0
MO simultaneously	0/10	6.0	100.0	0.0
MG simultaneously	0/10	5.5	100.0	0.0

Survival/Total—survived/total animals in the group; MST (mean survival time)—average survival time in days; SD—standard deviation; Mortality—percentage of mortality in the group; PI (protection index)—protection index. ^a^ Two-tailed Fisher’s exact test; * *p* < 0.05 vs. placebo; ** *p* < 0.001 vs. placebo; *** *p* < 0.0001 vs. placebo; ^#^ *p* < 0.05 vs. pleconaril; ^ns^ *p* > 0.05 vs. pleconaril; ^b^ one-way ANOVA (Bonferroni’s multiple comparison post-test); ^^^ *p* < 0.05 vs. placebo; ^^^^ *p* < 0.01 vs. placebo.

**Table 3:** Sensitivity to each compound in CPE inhibition tests of virus brain isolates from newborn mice.

**Group**	**IC_50_ Values (µM) of Viral Brain Samples (CVB1 Conn-5) Taken on Day**								
	**4**	**5**	**6**	**7**	**8**	**9**	**10**	**11**	**12**
PLECONARIL									
Placebo	0.119	0.159	0.131	-	-	-	-	-	-
Pleconaril	0.972	1.479	1.188	1.293	1.109	0.646	0.789	1.065	1.798
Pleconaril_day apart_	1.075	1.134	0.609	0.975	0.748	0.750	0.616	0.840	1.554
PO cons	0.163	0.052	0.174	0.112	0.043	0.031	0.051	0.044	0.091
PG cons	0.038	0.025	0.052	0.046	0.045	0.052	0.039	0.042	0.019
PM cons	0.150	0.192	0.076	0.028	0.049	0.043	0.030	0.034	0.078
PO simult	1.039	2.045	2.474	-	-	-	-	-	-
PG simult	1.600	2.957	-	-	-	-	-	-	-
PM simult	1.497	2.003	2.859	-	-	-	-	-	-
MDL-860									
Placebo	1.013	1.426	1.243	-	-	-	-	-	-
MDL-860	3.326	5.711	7.348	-	-	-	-	-	-
PM cons	1.815	1.950	1.312	1.262	1.217	1.953	1.103	0.742	0.903
MO cons	1.654	2.008	2.130	-	-	-	-	-	-
MG cons	1.907	2.367	-	-	-	-	-	-	-
PM simult	4.580	5.472	-	-	-	-	-	-	-
MO simult	3.902	4.133	-	-	-	-	-	-	-
MG simult	4.320	-	-	-	-	-	-	-	-
GUANIDINE HYDROCHLORIDE									
Placebo	329.7	353.1	302.1	-	-	-	-	-	-
Gua.HCl	337.2	411.5	-	-	-	-	-	-	-
PG cons	348.3	333.9	335.3	305.8	226.9	209.6	304.4	230.6	204.7
MG cons	356.7	389.6	-	-	-	-	-	-	-
PG simult	707.6	-	-	-	-	-	-	-	-
MG simult	715.2	-	-	-	-	-	-	-	-
OXOGLAUCINE									
Placebo	0.119	0.132	0.163	-	-	-	-	-	-
Oxoglaucine	0.143	0.265	0.429	-	-	-	-	-	-
PO cons	0.121	0.245	0.112	0.182	0.273	0.264	0.138	0.071	0.093
MO cons	0.110	0.240	0.310	-	-	-	-	-	-
PO simult	0.563	1.526	1.663	-	-	-	-	-	-
MO simult	0.410	1.350	-	-	-	-	-	-	-

- There was no animal alive; 100% mortality rate.

## Data Availability

The original contributions presented in the study are included in the article, further inquiries can be directed to the corresponding author.
